# Placebo Acupuncture Devices: Considerations for Acupuncture Research

**DOI:** 10.1155/2013/628907

**Published:** 2013-06-06

**Authors:** Dan Zhu, Ying Gao, Jingling Chang, Jian Kong

**Affiliations:** ^1^Department of Neurology, Dongzhimen Hospital, Beijing University of Chinese Medicine, No. 5 Haiyuncang, Dongcheng District, Beijing 100700, China; ^2^Department of Psychiatry, Massachusetts General Hospital, Harvard Medical School, Boston, MA 02129, USA

## Abstract

Determining an appropriate control for use in acupuncture research remains one of the largest methodological challenges acupuncture researchers face. In general, acupuncture controls fall under one of two categories: (1) sham acupuncture, in which the skin is punctured with real acupuncture needles either fully at nonacupoint locations or shallowly at acupoint locations or both and (2) placebo acupuncture, which utilizes nonpenetrating acupuncture devices. In this study, we will focus on non-penetrating placebo acupuncture devices (blunted-needle and nonneedle devices) that are currently available in acupuncture research. We will describe each device and discuss each device's validation and application in previous studies. In addition, we will outline the advantages and disadvantages of these devices and highlight how the differences among placebo devices can be used to isolate distinct components of acupuncture treatment and investigate their effects. We would like to emphasize that there is no single placebo device that can serve as the best control for all acupuncture studies; the choice of an acupuncture control should be determined by the specific aim of the study.

## 1. Introduction

 Over the past decades, acupuncture treatment has gained popularity in the Western world due to its therapeutic effect. However, studies have achieved contradictory results when using control treatments to test the true efficacy of acupuncture. Studies consistently show that both real and placebo acupuncture treatments confer significant benefits over no-treatment control conditions [1, 2], and while some studies have suggested that real acupuncture is significantly more effective than placebo acupuncture [3–5], others have failed to demonstrate the benefit of real acupuncture over placebo acupuncture [6–9]. Although the reasons for such contradictory results remain unclear, these results call for further investigation of sham/placebo controls in acupuncture research.

Placebo research has revealed several important variables related to acupuncture treatment that can be either modulated or held constant between verum (real) and placebo (control) conditions in order to test the effects of specific and nonspecific components of acupuncture. In general, acupuncture involves the insertion of needles into the body; thus, one of the components of verum acupuncture is skin penetration. While this particular component is not held constant between verum and non-penetrating acupuncture controls, there are several other variables that can be either held constant or modulated, which include sensory stimulation, dose (number of needles), acupoint location, practitioner-patient interaction, and treatment setting.

In acupuncture research, double-blinded randomized clinical trials (RCTs) serve as the gold standard when comparing the effects of a specific treatment to the effects of a placebo control. In acupuncture RCTs, it is ideal for the control treatment to be both physiologically inert and indistinguishable from the real treatment. Thus, an effective inert treatment in the control condition is essential in order for a double-blinded RCT to achieve a high level of scientific validity. Determining the proper inert control for an RCT designed to evaluate the efficacy of acupuncture is methodologically challenging for three main reasons.

1. It is difficult to create an inert control device that mimics both the visual appearance of the acupuncture treatment device and the method of needle insertion involved in acupuncture treatment.
2. It is challenging to develop an inert acupuncture device that can control for all nonspecific factors involved in an acupuncture treatment. The therapeutic effect of acupuncture relies on several nonspecific factors, including the ritual procedure of acupuncture administration, the patient-practitioner interaction, the nature of the illness, the treatment, and the treatment setting [10]. The microtrauma resulting from piercing the skin also induces a variety of nonspecific physiological responses involving the microcirculation, local immune function, and neurally mediated analgesic effects [11, 12].
3. It is challenging to make the acupuncturist who is directly administrating the acupuncture blind to treatment condition.

As a result, over the years, investigators have used a variety of controls in their studies to account for these challenges in terms of their own specific study aims. In acupuncture research, placebo controls for acupuncture studies fall under one of two categories: (1) sham acupuncture, in which the skin is punctured with real acupuncture needles either fully at nonacupoint locations or shallowly at acupoint locations, or both and (2) placebo acupuncture, which utilizes non-penetrating acupuncture devices. In this review, we will focus only on non-penetrating placebo acupuncture devices, including blunted needle and nonneedle devices that are currently available for use in acupuncture research. 

## 2. Placebo Acupuncture Devices

### 2.1. Blunted Needle Acupuncture Devices

#### 2.1.1. The Streitberger Device

**Figure 1:**
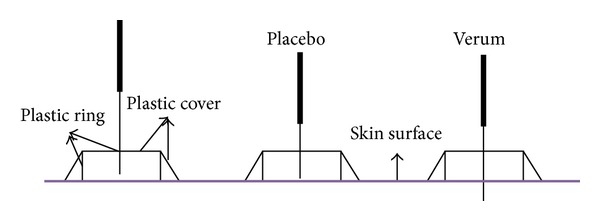
*The Streitberger device*. The Streitberger placebo device uses a short blunt needle within a thin handle. A plastic ring covered with a plastic sheath is used to keep the needle in place.

*Description. *In 1998, Streitberger and Kleinhenz [13] designed a blunted-needle placebo device, comprised of a copper handle and a stainless steel needle with a blunt tip designed to retract inside the handle (Figure 1). When the blunt tip is pressed against the skin, the patient feels a slight pricking sensation, which mimics the sensation elicited by a verum needle but does not actually puncture the skin. As the blunt tip is pressed onto the skin, the needle retracts into the handle, creating the appearance of penetration. This device is anchored in a plastic ring on the skin surface and held in place with surgical tape or plaster.

*Validation/Application*. The results from the first validation study of the Streitberger device [13] indicate that subjects in this study could not differentiate between verum acupuncture and the Streitberger device. In this crossover study, 60 healthy subjects, blinded to treatment condition, were asked to evaluate subjective ratings of acupuncture sensations using a visual analog scale (VAS) after both the placebo and verum acupuncture treatment.

Subsequently, the Streitberger device has been used as a control in acupuncture research [14–21] and clinical trials covering a range of indications [22, 23], resulting in a variety of findings. After the initial crossover experiment, Streitberger and colleagues conducted several trials in other clinical populations, the results of which indicated that the true efficacy of acupuncture may be specific to certain ailments [24]. For example, the investigators found verum acupuncture to be slightly more effective (*P* = .07) than placebo acupuncture in relieving postoperative nausea and vomiting prophylaxis in patients undergoing gynecological surgery but not for those undergoing breast surgery (*P* = .86) [24]. Results from another RCT conducted by Schneider and colleagues [25] suggest that the efficacy of acupuncture treatment for irritable bowel syndrome is primarily a placebo response. In one randomized trial [26], 52 males with rotator cuff tendinitis underwent 4 weeks of treatment, either with penetrating (verum) or non-penetrating (the Streitberger device) acupuncture, and were asked to rate their pain using the modified Constant-Murley shoulder outcomes scale (including pain, function, range of motion, and strength). The verum acupuncture group showed significantly more improvement in subjective pain ratings compared to the placebo acupuncture control.

#### 2.1.2. Park Device

**Figure 2:**
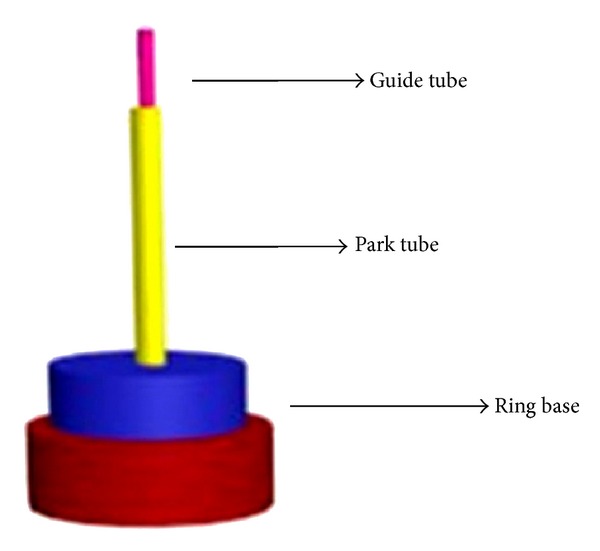
*The Park device*. The Park device is a placebo device that employs an oversize guide tube. The device adheres to the skin with double-sided tape. A second guide tube provides more stability, sliding to fit within the Park tube.

*Description.* Park and colleagues [27] invented a placebo device intended to stabilize the needle (either penetrating or blunted) in position with an additional plastic tube (Figure 2). The Park device consists of two tubes. The guide tube holds the needle perpendicular to the skin and is fitted within the Park tube, a larger tube fixed to the ring base, so that the standard guide tube slides freely within the Park tube. A silicon base rests under the ring base and adheres to the skin with double-sided tape. The acupuncturist positions the needle perpendicular to the skin and taps the handle of the needle in a precise imitation of the insertion of a real acupuncture needle. When a verum needle is used within the Park device, the needle is long enough to penetrate the skin. A placebo needle, in contrast, is too short to penetrate the skin through the Park device. After positioning the needle it may then be manipulated by lifting, thrusting, or rotating.

Unlike the Streitberger device, the Park device includes a Park tube that holds the placebo needle more firmly in place and facilitates lifting and thrusting, a common manual stimulation technique in acupuncture. Due to the fact that the guide tube is fixed to the skin, the Park device cannot be used to insert the needle at certain angles or depths. This limitation is problematic when using acupoints requiring shallow or horizontal insertion.

*Validation/Application.* The Park device was validated in two separate studies by Park and colleagues in 2002 [28]. The first study involved 58 acute stroke patients and the second involved 63 healthy acupuncture-naive volunteers. The purpose of these validation studies was to test the Park device in both a patient population and in healthy volunteers (aged 16 years and older) in order to assess whether the device was indistinguishable from verum acupuncture (study 1) and whether the device was “active” (i.e., whether it elicited sensations (deqi) specific to the needling action) when applied at the Hegu (LI4) point (study 2). The results of this RCT suggested that the Park device was both “indistinguishable” and “inactive" when employed. But there are some problems when using the Park device. For example, it may be more difficult to maintain participant blinding when using the Park device on traditional acupoints compared to nontraditional acupuncture points [29], or on the upper limb (i.e., Triple Energizer Meridian) acupoints compared to the lower limb acupoints (i.e., Bladder Meridian) [30].

Since the original validation studies, the Park device has been used in several acupuncture RCTs as a placebo acupuncture control [31–34]. For instance, in one RCT [35], the authors compared the efficacy of verum acupuncture (56 stroke patients) and the Park device (60 stroke patients) and they found that verum acupuncture was not superior to placebo treatment in terms of improvements in health-related quality of life after suffering a stroke.

#### 2.1.3. Japanese Device

**Figure 3:**
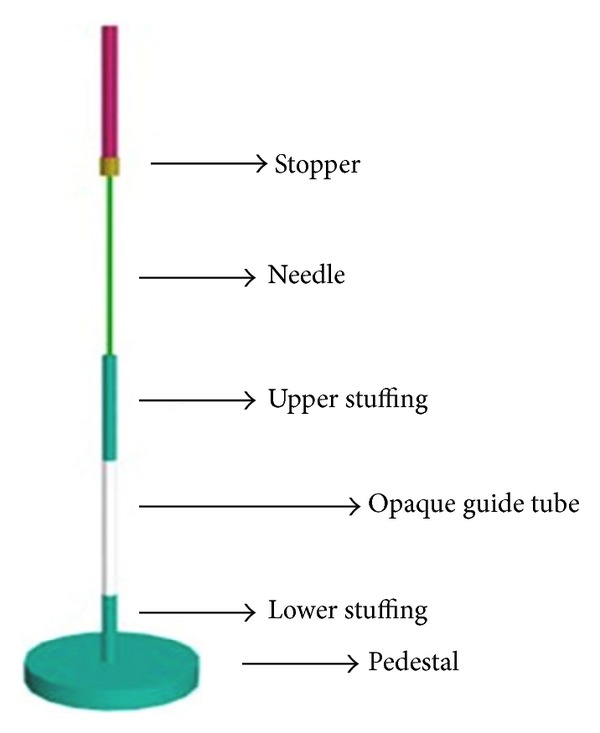
*The Japanese device*. The Japanese device is comprised of an opaque guide tube and an upper stuffing. The stopper prevents the needle handle from advancing further. In addition, the placebo contains stuffing at the bottom to provide a sensation similar to that of skin puncture and tissue penetration.

*Description.* The Japanese device, which employs a non-penetrating placebo needle (Figure 3), was designed by Takakura and Yajima for use in double-blind trials [36]. Like the needle of the Park and Streitberger devices, the tip of the placebo needle in the Japanese device makes non-penetrating contact with the skin. The Japanese device can be used with both verum and placebo needles and blinding of both patients and acupuncturists can be maintained. To our knowledge, the Japanese device is the only needle device that can maintain both patient's and acupuncturist's blinding. The verum and placebo needles used in the Japanese device differ in length. When a placebo needle is employed, the presence of the lower stuffing provides a substrate into which the needle can be inserted. When the verum needle is used, the sharp tip penetrates the skin. Inserting the needle into the lower stuffing of the placebo device produces a sensation similar to the sensation of skin being punctured for the patient and the sensation of tissue penetration for the acupuncturist, which allows both patient and acupuncturist to remain blinded to treatment condition. Both needles have a stopper that prevents the needle handle from advancing beyond the specified position of the penetrating needle or the non-penetrating needle. Due to fixed needle length and stuffing position, the Japanese device, like the Park device, is restricted in its ability to simulate traditional needle manipulation methods and various angles of insertion. This device requires custom manufacturing. Currently, it is only available for use by the inventor-investigators and awaits validation by other clinical trials.

*Validation/Application.* Investigations of the validity of the Japanese device suggest that it is quite indistinguishable from a verum device. In one study, the authors found that both experienced acupuncturists and acupuncture-experienced subjects made statistically equal numbers of correct and incorrect judgments of needle type (verum or Japanese device) after administration of treatments [37]. In another study, practitioners made more incorrect judgments than correct judgments [38] suggesting that this device was effective at maintaining practitioner's blinding.

#### 2.1.4. Foam Device

**Figure 4:**
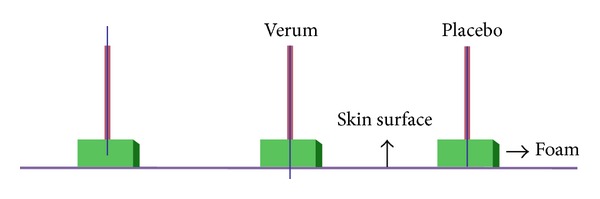
*Foam device*. The foam placebo (one example shown here) device uses a blunted needle inserted through a piece of elastic foam.

*Description.* There are several versions of the foam device used for placebo acupuncture treatment (see Figure 4 for one example). This device involves the use of a blunted needle and a cube of elastic foam for needle positioning. The acupuncturist must first fix the foam on a specified acupoint. In contrast to the verum acupuncture device, in which the sharp needle is inserted into skin through the foam, the placebo device holds the blunted needle within the foam. Compared with other devices, the foam device is simple and inexpensive. There are many variations of this device, so the specific design of the device must be taken into consideration. The Goddard device [39], for example, has no needle stopper, so it is the acupuncturist's responsibility to stop pushing the needle when s/he perceives that the placebo needle has touched the skin. Similarly, other foam devices require the acupuncturist to be careful during needle rotation not to advance the needle too far, in order to avoid skin contact entirely [40]. For all of the foam devices, the foam pad visually conceals the needle's point of entry, so that the subject cannot discern which technique is being used; it also helps to hold the needle in place so that it appears identical to real acupuncture needle positioning.

*Validation/Application.* In one randomized single-blind validation study, 49 healthy subjects were divided into two treatment groups: acupuncture group and placebo group. Goddard and colleagues [39] demonstrated that subjects were not able to differentiate between verum and placebo acupuncture. Placebo acupuncture was conducted by lightly pricking the skin with a shortened, blunted acupuncture needle through a foam pad, without penetrating the skin. 

In a study of 36 rheumatoid arthritis patients conducted by Tam and colleagues [40], investigators adhered a standard cube of foam material (2 cm × 2 cm × 2 cm) to the skin around the acupoint visible to patients. The recipient could not see the depth of the needle since the cube of foam hid the tip of the needles.

In another validation study using a crossover design [41], 32 healthy volunteers were randomly first assigned to either a verum acupuncture group or a placebo acupuncture group. After 30 minutes, the subjects received another modality of treatment. The main outcome measurement was a self-report questionnaire of deqi sensations. In this study, an adhesive patch made of two pieces of high-density foam, 13 mm in length, 5 mm in height, and 12.7 mm in width was perforated in order to hold a needle guidance tube. The guide tube sufficiently masked the needle from view such that subjects were not able to determine whether they were receiving real or placebo acupuncture when the patch was placed on the skin. In this study, a shortened needle with a blunted tip was used in order to avoid pricking the skin after tapping the needle. In this crossover study, there were no significant differences in ability to differentiate between the real and placebo needles before or after subjects received their second acupuncture condition. These results suggest that this method is credible for the subjects and constitutes a simple, inexpensive technique in use as a control in clinical research with a population of acupuncture-naive subjects.

#### 2.1.5. Other Nonpenetrating Needle Devices

*Description.* Multiple investigators have designed their own noninvasive needle-delivering apparatuses using blunted needles, toothpicks, or plastic guide tubes. These devices are simple, easy to manipulate, and do not require extra equipment. These control devices are designed to be as effective, inert, and indistinguishable as the devices that have preceded them; however, they do not mimic the appearance of the verum acupuncture devices with regard to needle insertion and therefore must be kept outside of the subject's visual range. They can be applied to the neck, back, and any other body region that the subject cannot see. If visible acupoints must be targeted, subjects' eyes must be covered to prevent unblinding. 

*Application.* The blunted needle device employs a needle, with the tip removed. The blunt tip of this specialized needle prevents the needle from penetrating the skin. The cut ends are manually smoothed with sandpaper under sterile conditions. The acupuncturist mimics needle insertion by applying the sparrow pecking technique (i.e., rapid stimulation of a single acupoint with the blunt tip of the needle) [4, 42]. 

Plastic guide tubes are also used as placebo devices, as they provide the sensation of acupuncture stimulation but do not penetrate the skin [43]. The plastic guide tube can also be combined with a blunted needle or toothpick to further provide the sensation of acupuncture stimulation [3, 7, 44–47].

Trials involving these devices are difficult to replicate because the devices are not standardized. Since the majority of these trials have not been precisely replicated (i.e., the devices are slightly different), the validity of the results of RCTs that use these devices remains to be tested.

### 2.2. Nonneedle Acupuncture Devices

#### 2.2.1. Transcutaneous Electrical Nervous Stimulation (TENS)

*Description.* Transcutaneous electrical nerve stimulation (TENS) is the application of a mild electrical current to the cutaneous nerve fibers using surface electrodes. It is characterized by current, pulse width, and frequency. The amplitude of the current is usually adjusted to just above or below sensory threshold. The duration of stimulation varies from a short period of time (e.g., 20 minutes) to continuous stimulation (e.g., 60 minutes or even longer). The placebo TENS device uses a nonfunctional TENS apparatus with no electrical stimulation. While the efficacy of verum TENS remains unknown, the placebo TENS device is an example of a double-blind placebo device that can act as a control to estimate the efficacy of the verum device because it is visually indistinguishable from the verum device. TENS differs entirely from needle acupuncture and thus the placebo TENS device may best serve as a control for verum TENS trials.

A new variation of TENS device worth noting is a special electrode designed for ear stimulation. It consists of two pair carbon-impregnated silicone electrodes fixed to one ear clamp; only one pair of the electrodes is connected to the electrical wire. Given that the electrode wiring is imbedded in the clamp, this design allows for subject and practitioner blinding [48].

*Validation/Application.* In an early double-blinded validation study on TENS-naive chronic low back pain patients, real TENS was compared to sham TENS. The results showed that every patient in the real TENS group believed the unit was functioning correctly with varying degrees of certainty. In the sham TENS group, 84% patients believed they have a functioning unit, with significant lower certainty level [49].

Several subsequent clinical trials have employed placebo TENS to investigate the efficacy of verum TENS. In a study of chronic back pain, a 2 × 2 factorial design was used to compare TENS, placebo TENS, exercise, and no exercise. No superior benefit was found for verum TENS over placebo TENS using a VAS of pain ratings and other clinical outcome measures [50].

In another study, verum TENS versus placebo TENS was studied in patients with multiple sclerosis and chronic low back pain. After correcting for multiple comparisons, there were no significant differences on pain visual analog scale and other self-evaluation scales between the verum and placebo TENS groups [51].

In one study, investigators studied the efficacy of TENS compared to placebo TENS and a control no-treatment group in a population of patients with chronic low back pain. Average pain ratings collected immediately before and after each treatment demonstrated a significant reduction in pain in both the verum and placebo TENS groups. Pain intensity was reduced significantly more for those in the TENS group compared to the placebo TENS group. Additionally, investigators studied the additive effects of 10-week treatment (administered twice weekly) and found that TENS but not placebo TENS demonstrated a reduction in pain intensity over the first 16 treatments. Similarly, verum TENS was more effective than placebo TENS in maintaining pain reduction one week after the last treatment. The benefit in pain reduction continued for 3 and 6 months after completion of the study regardless of whether subjects received verum or placebo TENS treatment. The no-treatment control condition, in contrast, demonstrated no natural improvement in pain over the same course of time [52].

#### 2.2.2. Laser Acupuncture

*Description.* Laser acupuncture is defined as the stimulation of traditional acupuncture points with low-intensity, nonthermal laser irradiation. Verum laser acupuncture produces a wavelength of infrared laser light. Both verum and placebo laser acupuncture are manufactured with visual red light and acoustic signal. 

*Validation/Application. *In 2001, Irnich and colleagues [53] were the first investigators to adopt the placebo laser acupuncture device. They used placebo laser acupuncture as the inert control in a study that compared conventional massage with acupuncture (verum needle and placebo laser acupunctures) for the treatment of chronic neck pain. The placebo laser acupuncture was performed with an inactive laser pen, which produced a red light with no infrared properties. The results from this study suggest that needle acupuncture is significantly more effective than massage and equally as effective as a short-term treatment for patients with chronic neck pain, indicating that placebo laser acupuncture might share some of the same nonspecific effects with needle acupuncture.

The validity of the placebo laser acupuncture as a general acupuncture control was thoroughly discussed in an article written by Irnich et al. [54]. In another study they also investigated the validity of the placebo laser acupuncture device as a laser acupuncture control. The results of this randomized, double-blind crossover study suggested that placebo laser acupuncture produces the same nonspecific effects as laser acupuncture. Additionally, there were no significant differences between the efficacy ratings of acupuncture-experienced and acupuncture-naive subjects in this study. Neither the subject nor the treating acupuncturists were able to distinguish between the real and placebo laser devices. Over the years, this device has been applied in several trials to treat diseases such as acute tonsillitis, and pharyngitis, vasomotor rhinitis and whiplash injuries [55–58].

## 3. Discussion

 Finding a proper control remains a primary methodological concern in acupuncture research. Traditionally, two main categories of acupuncture control have existed: (1) sham acupuncture, which involves the use of real needles that puncture the skin and (2) placebo acupuncture, which is non-penetrating. Sham acupuncture utilizes penetrating needles that are either applied fully to nonacupoints or shallowly to acupoints or both [8]. But the problem is that it can be difficult to find a noninfluential site on the skin that is not near other acupoint, and shallow needling can resemble some traditional Chinese acupuncture techniques. Several studies, however, have indicated that sham acupuncture can also produce a therapeutic response and elicit neurobiological responses at various levels in the central nervous system [59].

Thus, a placebo acupuncture device may be a more appropriate control for verum acupuncture because it minimizes the physiologic response and is relatively inert. In this paper, we have described a range of placebo acupuncture devices currently used in acupuncture research.  Table 1 lists the relative advantages and disadvantages of the devices, as well as the common and unique aspects of the devices as discussed below. With this table, we seek to highlight some of the considerations investigators should take when designing their placebo-controlled acupuncture studies.

Placebo acupuncture is generally noninvasive. The blunted needles used in these devices are relatively inert and indistinguishable from real acupuncture needles. The Streitberger, Park, and Japanese devices are three of the most commonly used placebo needle devices in acupuncture research. The Japanese device is the only needling device that can be employed in double-blinded trials. All three of these devices are used in a similar manner; a blunted needle is inserted, touches the skin, and is retracted. These three devices, while standardized and validated, are not widely available for researchers worldwide since they are custom fabricated. Aside from the Streitberger device, all other placebo devices have physical limitations with regard to their ability to facilitate a range of manipulation methods, such as shallow or horizontal insertion. They are also not effective for all acupoint types and acupuncture positions. The foam device, simple toothpick, and blunted needle device are not standardized and thus cannot be validated. Since these devices have not been validated, the quality of the results from RCTs using these devices remains unconfirmed. 

All placebo needle devices involve contact with the skin. Physical contact with the surface of the skin may provide sensory stimulation, indicating that even blunted needle devices are not entirely inert and may elicit a therapeutic effect of their own. For instance, Han and Lund et al. noted that the sensation produced by a needle tip “touch” was substantial [60] and it may activate parts of the peripheral nervous system [61]. Thus, using blunted needle devices on nonacupoints in acupuncture studies might make the placebo acupuncture relatively inert.

The response to placebo acupuncture treatment is due to a variety of factors, including the presence or absence of deqi sensations. The deqi phenomenon is a complex set of physiological sensations associated with acupuncture treatment [14, 62]. Those who have experienced acupuncture deqi sensations prior to treatment are often cognizant of the absence of deqi when they receive placebo acupuncture treatment. For instance, in one single-blind randomized, crossover pilot study [63] that involved patients with chronic pain receiving both verum and placebo acupuncture treatment, subjects who received the placebo treatment first thought that they were receiving verum treatment after one single treatment. However, after the second treatment, nearly 40% of those same subjects were able to detect a difference between the two needles. Thus, it is also important to measure the deqi sensation in acupuncture clinical trials even when sham devices are applied. In light of this finding, some investigators [46] choose to exclude patients with previous acupuncture experience. 

Similar to other longitudinal treatment studies involving placebo treatments, maintaining patient's blinding throughout a treatment study involving multiple acupuncture treatments can be difficult. All blunted needle placebo acupuncture devices can be useful in short-term, acute intervention trials. However, in conditions requiring long-term treatment, maintaining patient's blinding may be difficult due to the subjects' natural curiosity and/or motivation to learn more about the type of treatment they have been receiving, which can lead to unblinding (e.g., patients may talk to each other, read about acupuncture, or go to another acupuncturist). It is important for investigators to assess whether or not the subject blinding can be maintained through the end of the study.

Unlike verum acupuncture, some placebo devices require tape or foam for successful application of the placebo treatment. Devices that require tape or foam may induce allergic reactions and thus may not be tolerated by all subjects. In addition, there is risk of infection if the needle were to be inserted through the tape and thus it is necessary to use sterile techniques. Additionally, these devices may not be suitable for all acupoints, such as points on the scalp, fingers, and toes that cannot provide a flat surface for tape or foam.

“Nonneedle” placebo acupuncture devices (TENS and laser acupuncture) have their own set of common considerations. Neither placebo TENS nor laser acupuncture provide repeated needle stimulation of the skin and thus are relatively physiologically inert. Nonneedle placebo devices are also effective for both acupuncture-experienced and acupuncture-naive subjects. The major disadvantage of nonneedle devices like TENS and laser acupuncture is that they differ from verum needle acupuncture in design and concept, as well as in context and culture. Therefore, placebo TENS and placebo laser acupuncture may only be considered to serve as valid placebo controls in TENS and laser acupuncture studies, respectively.

Finally, we would like to emphasize that this paper is developed to aid investigators in designing studies that test and explore the efficacy and mechanism of acupuncture and to facilitate the selection of appropriate acupuncture placebo devices. The most appropriate placebo acupuncture devices are those that are the most indistinguishable and inert in consideration of the specific design on the study.

## Figures and Tables

**Table 1:** Considerations for use of placebo devices in acupuncture research.

Types of device	Considerations									
	Visually indistinguishable	Somato-sensation	Constraint of needle insertion	Commercial availability	Self-made	Modality specific	Price	Reusable	Appropriate for double-blind study design	Appropriate for crossover study design
Blunted needles devices										
The Steitberger device	*√*			*√*		*√*	$6.3 per needle			
The Park device	*√*		*√*	*√*		*√*	$2.9 per needle			
The Japanese device	*√*		*√*			*√*	NA		*√*	
Foam device	*√*		*√*		*√*		NA	*√*		
Others	*√*				*√*		NA	*√*		

Nonneedle devices										
Placebo TENS	*√*		NA	*√*		*√*	$59 to $260 per unit	*√*	*√*	*√*
Placebo laser acupuncture	*√*	*√*	NA	*√*		*√*	$hundred to thousand per unit	*√*	*√*	*√*

Prices according to Google Shopping. The actual prices may vary across vendors.
